# Discrete components of stress impact trajectories of childhood irritability but not adolescent mental health outcomes

**DOI:** 10.1002/jcv2.70153

**Published:** 2026-08-09

**Authors:** Mariah DeSerisy, Huiyu Yang, Jacob W. Cohen, David Pagliaccio, Julie B. Herbstman, Bradley S. Peterson, Frederica P. Perera, Virginia Rauh, Amy E. Margolis

**Affiliations:** ^1^ Department of Psychiatry and Behavioral Health The Ohio State University Columbus Ohio USA; ^2^ Department of Psychiatry Vagelos College of Physicians and Surgeons Columbia University New York New York USA; ^3^ Division of Child and Adolescent Psychiatry New York State Psychiatric Institute New York New York USA; ^4^ Department of Environmental Health Sciences Mailman School of Public Health Columbia University New York New York USA; ^5^ Columbia Center for Children's Environmental Health Mailman School of Public Health Columbia University New York New York USA; ^6^ Institute for the Developing Mind Children's Hospital Los Angeles Los Angeles California USA; ^7^ Department of Psychiatry Keck School of Medicine at the University of Southern California Los Angeles California USA; ^8^ Heilbrunn Department of Population and Family Health Mailman School of Public Health Columbia University New York New York USA; ^9^ The Child Mind Institute New York New York USA

**Keywords:** adolescent anxiety mental health, childhood mental health, familial stress, intimate partner violence, irritability

## Abstract

**Background:**

Exposure to stressors during early childhood has been linked to irritability and subsequent mental health problems; however, little work has examined the unique and combined effects of distinct stress exposures on trajectories of childhood irritability or adolescent mental health symptoms.

**Methods:**

Six components of family stress were assessed via maternal report at child age 5. Irritability was measured longitudinally at ages 7, 9, and 11. Adolescents self‐reported on anxiety, depression, and externalizing symptoms at age 13–20. Latent growth mixture modeling examined classes of irritability. Bayesian kernel machine regression (BKMR) examined unique and shared effects of stress components on class assignment and to continuously measured irritability at individual age points (*N* = 472). Causal mediation tested if irritability mediated any stress‐related mental health problems.

**Results:**

Latent growth mixture modeling identified high and low irritability classes. A positive joint mixture effect of stressors on irritability was detected, with additive contributions from intimate partner violence, material hardship, perceived stress, and maternal demoralization. Intimate partner violence independently predicted high irritability over time and irritability at age 7, and maternal perceived stress independently predicted irritability at age 11. Neither childhood irritability nor early stress predicted self‐reported adolescent mental health symptoms.

**Conclusion:**

Early exposure to familial stressors are risk markers for childhood irritability. Interventions targeting intimate partner violence and co‐occurring stressors could help mitigate persistent irritability, a well‐known public health concern related to children's development.

## Key Points

**What's known?**

- Familial stressors are linked to childhood irritability and later mental health problems, such as anxiety and depression.

**What's new?**

- This study finds exposure to intimate partner violence at age 5 is associated with persistent irritability, even after accounting for other stressors. When occurring conjointly, a mixture of stressors also elevated irritability risk.

**What's relevant?**

- Intimate partner violence is a modifiable risk, highlighting opportunities for targeted intervention.

## Graphical abstract

Thick red line indicates positive mixture effect of stressors predicting high irritability class. Maternal demoralization, material hardship, maternal perceived stress, and intimate partner violence contributed to the mixture effect (thick borders on boxes). Light red lines indicate that intimate partner violence independently predicted high irritability over time and irritability at age 7, and maternal perceived stress independently predicted irritability at age 11. Gray dashed lines indicate that neither childhood irritability nor early stress predicted self‐reported adolescent mental health symptoms.

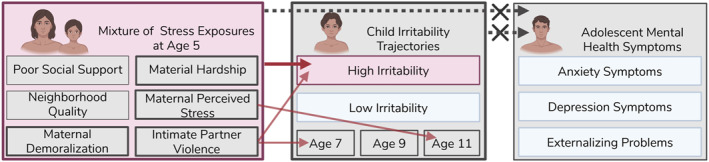

## INTRODUCTION

Irritability describes proneness to anger and is characterized by chronically negative moods and disruptive behaviors (S. C. Evans et al., 2020; Moore et al., 2019). Clinically significant irritability affects as many as twenty percent of youth and ranks among the leading reasons children and families seek mental health treatment (Copeland et al., 2015; S. C. Evans et al., 2021; Mayes et al., 2016; Peterson et al., 1996). Irritability is also a marker of risk for future psychopathology, in particular adolescent mood and anxiety disorders (Dougherty et al., 2015; Sorcher et al., 2022), which are fueling the current crisis in child mental health (Benton et al., 2021). Critically, even in non‐clinical samples, elevated irritability is associated with greater functional impairment (Copeland et al., 2015), pointing to the importance of investigating the etiology of irritability to identify key targets for public health intervention and, ultimately, reshape symptom trajectories for irritable youth.

In prospective longitudinal samples, childhood irritability has been independently linked with many individual types of stressful experiences including parental mental health issues (particularly depression), harsh parenting practices, and family dysfunction (Smith et al., 2019; Wiggins et al., 2014; Yu et al., 2022). Similarly, living in low income contexts also increases risk of irritability, possibly through exposure to stressful experiences (Lengua, 2006; Piotrowska et al., 2015, 2019). In cohorts enriched for trauma exposure (Bielas et al., 2016; Cotter et al., 2024; Grasser et al., 2024; Henriksen et al., 2021; Hodgdon et al., 2021; McCoy et al., 2016; Zhan et al., 2024) or other forms of psychopathology, such as attention deficit hyperactivity disorder, anxiety, or depression (Hartman et al., 2019; Kessel et al., 2021; McCoy et al., 2016; Wakschlag & Keenan, 2001; Zendarski et al., 2022), exposure to psychosocial stress and family violence also predicts elevated irritability. Given that stressful life experiences are associated with irritability in both clinical and nonclinical samples, family stress and trauma exposure may represent critical targets for both prevention of and intervention on clinically significant irritability.

Despite evidence of the importance of independent stressors to irritability in studies examining single stress exposures, many studies examining multiple stressors only investigate the effects of total stress exposure on irritability, that is, number of adverse childhood experiences (Felitti et al., 1998), limiting the ability to understand which specific stressors contribute to irritability. Three extant cross‐sectional studies show that exposure to interpersonal violence, and in particular psychological violence, is associated with increased irritability. Two studies recruited trauma‐exposed, treatment seeking youth, limiting their generalizability (Cotter et al., 2024; Grasser et al., 2024). The third study leveraged a community sample of adolescents (*N* = 7422, 13‐to‐19‐year‐olds) and showed that each of ten traumatic events (including physical and sexual violence) was independently associated with irritability. This study, however, did not examine changes in irritability over time, nor did it test the effects of multiple exposures concurrently, thereby limiting the understanding of how combinations of stressors contribute to irritability (Henriksen et al., 2021). Furthermore, only Cotter et al. (2024) examined the effect of traumatic events in conjunction with other non‐traumatic psychosocial stressors (e.g., poverty).

Despite substantial evidence that irritability is a risk marker for future psychopathology, and that exposure to stress increases risk for both irritability and psychopathology, the pathways through which stress influences adolescent psychopathology remain complex. In one longitudinal case‐control study of irritability, negative life events moderated associations between irritability and anxiety symptoms among the healthy youth only (Archer et al., 2024); however, no studies have yet specifically investigated if irritability is on a pathway from stress to psychopathology. The current study examines the independent and joint effects of multiple psychosocial stressors (material hardship, maternal perceived stress, intimate partner violence, lack of social support, neighborhood quality, and nonspecific maternal distress) on trajectories of children's irritability and the later adolescent mental health symptoms in an epidemiological birth cohort of Black and Latiné mothers and children (*N* = 472). Importantly, we include prospective reporting of stressors which may overcome memory biases that are potentially introduced in studies that rely on retrospective recall of experiences (Jivraj et al., 2020; Talari & Goyal, 2020). We hypothesized that greater stress exposure contributes to higher irritability across childhood and, based on prior findings, that exposure to violence would be a particularly salient contributor (Cotter et al., 2024; Grasser et al., 2024; Henriksen et al., 2021). We further hypothesized that irritability would mediate the link from stress to adolescent psychiatric symptoms.

## MATERIALS AND METHODS

### Participants

Data used in the current study was collected as a part of an ongoing longitudinal prospective birth cohort study conducted at the Columbia Center for Children's Environmental Health (CCCEH); detailed demographics and recruitment information have been previously published (Perera et al., 2006). Briefly, participants were Black and Latiné women recruited between 1998 and 2006 through prenatal care clinics in Northern Manhattan and Washington Heights in New York City, United States. Recruited women (ages 18–35 years) were non‐users of tobacco products or other substances, and were free of diabetes, hypertension, or known HIV, and had initiated prenatal care by the 20th week of pregnancy. A total of 727 mother‐child dyads were recruited. The cohort study is approved by the Institutional Review Board of Columbia University; initial approval occurred around 11/01/1997 and has been reviewed annually since that date. The most recent annual renewal was approved on 5/5/2026. Mothers provide informed consent at every study visit. Children provide assent beginning at age 7.

Childhood irritability scale development and latent class analyses included all cases with any available irritability data (*N* = 511). All remaining analyses required complete cases (*N* = 472); cases with missing data were deleted listwise.

### Study timeline

Longitudinal study visits began in the third trimester of pregnancy and occurred approximately every 2 years thereafter. The current study leverages behavioral data collected during four visits: “age 5” (range: 4–6 years; mean age = 4.46 years), “age 7” (range: 6–8 years; mean age = 6.54 years), “age 9” (range: 8–11 years; mean age = 8.58 years), and “age 11” (range: 10–13 years; mean age = 10.56 years). Measures of children's irritability were collected during children's age 7, 9, and 11 year visits; stress measures were collected during children's age 5 study visit. Adolescent self‐report of anxiety, depression, and externalizing symptoms were measured once. Anxiety and depression symptoms were measured at mean age 13.68 years and externalizing problems were measured at mean age 16.86 years.

### Discrete familial stressors

At child age 5, mothers completed a structured interview assessing demographics and six domains of maternal experiences of stress during the child's early life. As such, we refer to them as familial stressors because they reflect the shared experience of stress between mother and child (Bush et al., 2017; Rudd et al., 2022). Domains of stress were obtained from several published scales examining: (1) past year material hardship (Material Hardship Interview; Mayer & Jencks, 1989); (2) past month maternal perceived stress (Perceived Stress Scale; Cohen et al., 1983); (3) intimate partner violence experienced during the child's lifetime (Conflict Tactics Scales, revised; Straus et al., 1996); (4) lack of current social support (Interpersonal Support Evaluation List; Cohen et al., 1985, 2000); (5) current neighborhood quality (Neighborhood Quality Evaluation Scale; Kim et al., 2008; Sampson & Raudenbush, 1999; Sampson et al., 1997) and (6) past year nonspecific maternal distress or demoralization (Psychiatric Epidemiology Research Instrument–Demoralization; Dohrenwend et al., 1980). A description of the constructs measured by the scales and the individual scale items are presented in Supporting Information S1: Appendix S1. In prior work, these scales demonstrate good reliability and psychometric properties (Dohrenwend et al., 1980; Kim et al., 2008; Straus, 2017; Vega & O’Leary, 2007).

Polychoric alpha assessed internal consistency in the current sample (Flora, 2020; Gadermann et al., 2012; Zumbo et al., 2007), and values were interpreted according to Tavakol & Dennick, 2011 as: 0.70‐0.80 acceptable, 0.80‐0.90 good, >0.90 excellent. All scales demonstrated acceptable internal consistency (Supporting Information S1: Table S1). All scales were z‐score standardized with higher scores indicating more stress exposure. Outliers |Z = ±3| were winsorized to the next non‐outlier value. Correlations between stress scales were high, supporting a mixture‐based approach (Supporting Information S1: Table S2).

### Childhood irritability

Parent reported irritability in children ages 7–11 was quantified using a subset of item (Table 1) from the Children's Behavior Checklist (CBCL; Achenbach TM, 2001) and Conners Parent Report Scale (CPRS; Conners et al., 1998; *N* = 511). Item‐level data were *z*‐scored with higher scores indicating more irritability. Responses were averaged within each scale and then averaged across parent‐report scales to create a single composite score (range = −1–3) of child irritability at each age.

**TABLE 1:** Items included in irritability score.

Test	Age (years)	*N*	Relevant items
Child behavior checklist	7	511	86. Irritable mood
	9	462	87. sudden changes in mood
	11	367	95. hot temper
Conners parent rating scales	7	504	1. Angry and resentful
	9	467	21. Loses temper
	11	366	31. Irritable
			47. Temper outbursts
			68. Demands must be met immediately—easily frustrated
			77. Mood changes quickly and drastically
			78. Easily frustrated in efforts

To validate the single irritability composite, fit indices from confirmatory factor analysis, internal consistency, discriminative validity, and test‐retest reliability were examined. Confirmatory factor analysis model fit was assessed using root mean square error of approximation (RMSEA), comparative fit index (CFI), and the standardized root mean square residual (SRMR), as these are recommended for smaller sample sizes (*N* < 500; Maas & Hox, 2005; Schumacker & Lomax, 2015). To indicate a valid scale, the RMSEA should be close to zero with a significance value > 0.05 (Cudeck, 1993; Jöreskog & Sörbom, 1993), CFI >0.90 (Browne & Cudeck, 1992; Little, 2013), and SRMR <0.08 (Pavlov et al., 2021). We compared a single factor model to two‐factor, bifactor, hierarchical, and two‐factor with fixed covariance solutions at each age point. CBCL and CPRS items were treated as ordinal categorical variables; models were fitted using a Weighted Least Square Mean and Variance‐adjusted (WLSMV) estimator. Latent variables were standardized (Bollen, 1989). Satorra–Bentler scaled chi‐square difference test and fit metrics compared the single and two‐factor models. Discriminant validity was assessed via the heterotrait–monotrait ratio (HTMT; Henseler et al., 2015) where values > 0.85 indicate poor discriminant validity between factors. Bifactor models tested if the CBCL and CPRS factors were empirically distinguishable. Cronbach's Hierarchical omega values > 0.80 and ECV >0.70 are often considered strong support for a dominant general factor (Rodriguez et al., 2016).

The single factor and general bifactor scores were compared using Pearson correlations to test how similarly these captured the same underlying construct. Polychoric alpha was used to assess internal consistency of the optimal factor solution (Supporting Information S1: Table S1). Test‐retest reliability was investigated via Pearson's correlations and intraclass correlations (ICC) at all three timepoints with ICC interpreted as: between 0.5 and 0.75 moderate, and 0.75–0.9 good (Koo & Li, 2016). Factor scores were used in all subsequent analyses.

### Adolescent mental health symptoms

Adolescent mental health symptoms were measured using the three scales described below. All symptom scales were *z*‐scaled prior to inclusion in the models.

*The Revised Children's Manifest Anxiety Scale* (RCMAS; Reynolds & Richmond, 1978, 1985; K. S. White & Farrell, 2001) is a 37‐item measure of anxiety symptoms. Items are rated as present or absent. Total sum score scores include 28 anxiety items, ignoring the 9 social desirability items. Scores range from 0 to 28. Total scores above 19 may indicate clinically significant anxiety (Stallard et al., 2001). Internal consistency is presented in Supporting Information S1: Table S1.

*The Center for Epidemiologic Studies Depression Scale* (CES‐D; Radloff, 1977) is a 20‐item self‐report of depression symptoms validated for individuals older than six years. Items are rated on a 0–3 scale indicating symptom frequency (rarely or none to most or all of the time). Positively worded items are reverse scored. Sum total scores range from 0 to 60, with scores above 15 indicating significant levels of depression symptoms (Weissman et al., 1980). Internal consistency is presented in Supporting Information S1: Table S1.

*The Youth Self‐Report of the Children's Behavior Checklist* (YSR; Achenbach & Rescorla, 2001) is a 112‐item self‐report measure of socioemotional functioning for youth ages 11–18 years old but has been used as an epidemiologic research tool for young adults up to age 22 (e.g., Ferdinand et al., 2004). The YSR derives several subscales, of which the Externalizing Problems subscale was used in the current analyses. The Externalizing Problems subscale contains items reflecting rule‐breaking and aggressive behavior, such as “I destroy things belonging to others” and “I disobey my parents.” Items are rated 0–2, not true to very true or often true. Subscale scores are generated by summing across 32 relevant items and converting to t‐scores based on youth age and sex. T‐scores between 60 and 64 indicate risk for problem behaviors; *t*‐score ≥65 indicate clinically significant externalizing problems. Internal consistency is presented in Supporting Information S1: Table S1.

### Analytic plan

We derived longitudinal trajectories of irritability via growth mixture modeling using R function “hmle” (Proust‐Lima C PV, Diakite A, Liquet B, Proust MC, 2016). Growth mixture modeling is a person‐centered, probabilistic approach for identifying latent subpopulations and characterizing heterogeneity in trajectories (Ram & Grimm, 2009; Sijbrandij et al., 2019). We tested 1–5 class solutions using linear and quadratic specifications; both theoretical and statistical fit were considered (Ram & Grimm, 2009). Fit statistics included maximum log‐likelihood (similar to AIC for nonlinear link functions), Bayesian Information Criteria (BIC), sample size‐adjusted BIC [SABIC], entropy, interpretability of classes, and class size. We required that >10% of the sample be included in each of the latent classes.

Bayesian kernel machine regression (BKMR) analyses were examined as a nonparametric framework to estimate three outputs: (1) the total effect of the stress “mixture” on irritability trajectory class assignment (i.e., the combined effect of all of the stressors), (2) the effect of each stressor on irritability while holding effects of other stressors constant and taking into account the shape of the association between stressor and irritability [for example, linear, nonlinear, etc.]), (3) bivariate or interaction effects between stressors on irritability. See Supporting Information S1: Appendix S1 for details. BKMR was implemented in R (bkmr; Bobb, 2022). Follow‐up analyses examined BKMR on irritability total scores at each age point independently. Variables used in mediation analyses were selected based on posterior inclusion probabilities (PIP) > 0.50, as these indicate relative importance to the mixture (Barbieri & Berger, 2004; Nunez et al., 2021); those with PIP >0.5 for the trajectory and two of three age point models were retained. Finally, mediation analyses assessed a potential pathway from social stressors to adolescent mental health symptoms (anxiety, depression, externalizing) via childhood irritability. This was implemented in R (“MASS” package) using simulation‐based approach (5000 iterations) to estimate the direct, indirect, and total effects. First, to approximate total stress effects, joint mixture effects were extracted from BKMR models. Next, individual stressors associated with longitudinal irritability class were entered into the mediation analyses. Each model included irritability class as the mediator and thus a logistic regression from stress to irritability. Linear regressions examined the paths from stressor and irritability to symptoms. Follow up analyses examined direct effects of stressors or irritability on adolescent mental health outcomes via FDR‐corrected linear regression, controlling for confounders. We also computed Bayes factors (BF_10_; BayesFactor package; Morey, n.d.) to test if non‐significant findings indicated the absence of results (Kass & Raftery, 1995; Lakens et al., 2020).

Covariates known to be associated with familial stressors and/or irritability were included in all models testing associations of stress with irritability, including child sex at birth (Cho et al., 2015; DiPietro & Voegtline, 2017), maternal years of education as a proxy for socioeconomic status (Barrett & Turner, 2005; Perlis et al., 2024), maternal age at child's birth (D’Onofrio et al., 2009; van Roode et al., 2017), and quality of the home environment (G. W. Evans et al., 2001).

## RESULTS

### Participants

Table 2 presents demographic information of the included sample (See Supporting Information S1: Appendix S2, Table S3, Figure S1 for more details).

**TABLE 2:** Demographic characteristics.

	Mean (SD)/N (%)
Sex (% male)	243 (47.83)
Latiné (%)	314 (62)
Black (%)	195 (38)
Maternal years of education at prenatal visit	11.9 (2.16)
Maternal age at child birth	24.95 (4.97)
Maternal demoralization at child age 5	1.01 (0.67)
Intimate partner violence at child age 5	0.16 (0.21)
Neighborhood quality at child age 5	0.47 (0.32)
Quality home environment	39.28 (6.31)
Age 7 irritability	0.11 (0.18)
Age 9 irritability	0.14 (0.17)
Age 11 irritability	0.13 (0.16)
Adolescent anxiety (RCMAS total score)	8.88 (6.00)
Adolescent depression (CESD total score)	16.89 (6.50)
Adolescent externalizing problems (YSR externalizing problems T‐score)	49.56 (9.27)

Abbreviations: %, percent; CESD, Center for Epidemiologic Studies Depression Scale; RCMAS, Revised Children's Manifest Anxiety Scale; SD, standard deviation; YSR, Youth Self‐Report of the Children's Behavior Checklist.

### Childhood irritability scale

Figure S2 shows item correlations across all time points. A single factor solution was selected for use in all analyses given the strong unidimensional factor structure indicated by psi, omega, HTMT, ECV and goodness‐of‐fit indices which contrast with the two factor solution indicated by the Satorra‐Bentler test (Table 3, Supporting Information S1: Appendix S2, Tables S4 and S5, Figures S3 and S4).

**TABLE 3:** Model fit indices for one‐ and two‐factor confirmatory factor analyses.

Model	Χ^2^	df	CFI	RMSEA	RMSEA CI	SRMR	Ψ	Δ Χ^2^	Δdf	p
Age 7										
Single‐factor	62.322	35	0.996	0.044	0.025–0.061	0.057				
Two‐factor	52.824	34	0.998	0.037	0.015–0.055	0.049	0.881	9.801	1	0.002
Age 9										
Single‐factor	82.952	35	0.992	0.060	0.043–0.077	0.071				
Two‐factor	68.703	34	0.994	0.052	0.034–0.069	0.062	0.853	14.219	1	0.0002
Age 11										
Single‐factor	56.257	35	0.996	0.044	0.021–0.065	0.060				
Two‐factor	53.819	34	0.996	0.043	0.019–0.064	0.060	0.941	3.596	1	0.058

Abbreviations: Δ, change in; CFI, comparative fit index; CI, confidence interval; df, degrees of freedom, RMSEA, root mean square error of approximation; SRMR, standardized root mean square residual.

### Latent growth mixture modeling

**FIGURE 1:**
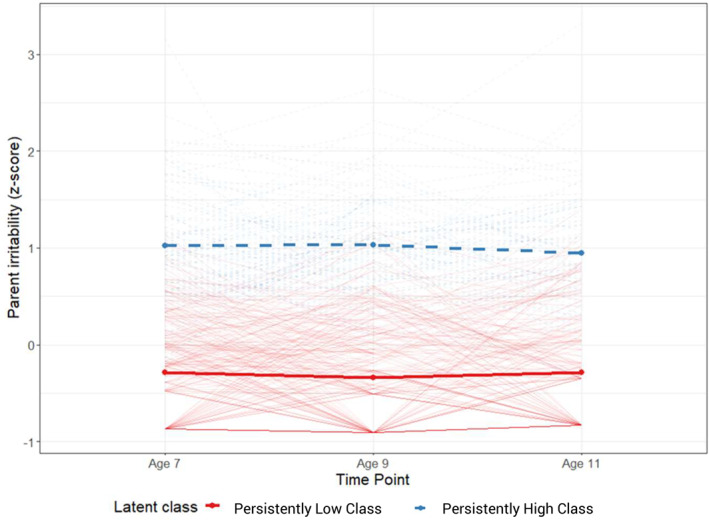
Latent growth mixture modeling trajectories of parent‐reported irritability. Pale lines represent individual subject trajectories. Dashed blue lines represent the persistently high irritability class (29.204%, *N* = 132) and solid red lines represent persistently low irritability class (70.796%, *N* = 320).

The two class linear model was the best fitting model (Table 4; Figure 1) showing a “persistently low” irritability class (70.796%, *N* = 320) and a “persistently high” irritability class (29.204%, *N* = 132).

**TABLE 4:** Results of latent growth mixture modeling.

Class	Max log‐ likelihood	BIC	SABIC	Entropy	Percent in class 1	Percent in class 2	Percent in class 3	Percent in class 4	Percent in class 5
Linear models									
1	−1192.32	2415.201	2399.333	1	100				
**2**	**−1171.19**	**2391.298**	**2365.909**	**0.682**	**70.796**	**29.204**			
3	−1171.19	2409.639	2374.729	0.354	0	67.478	32.522		
4	−1171.19	2427.98	2383.549	0.284	65.708	0	0	34.292	
5	−1171.19	2446.321	2392.369	0.255	0	61.947	0	0	38.053
Quadratic models									
1	−1192.140	2420.962	2401.920	1	100				
2	−1170.417	2401.972	2370.235	0.682	29.425	70.575			
3	−1159.740	2405.071	2360.640	0.669	28.982	56.195	14.823		
4	−1159.740	2429.525	2372.400	0.467	35.177	0	50.000	14.823	
5	−1135.735	2405.971	2336.151	0.633	10.398	10.398	45.354	12.611	21.239

*Note:* Bolded values reflect the best fitting model.

Abbreviations: BIC, Bayesian Information Criteria; SABIC, sample size‐adjusted BIC.

### BKMR

**FIGURE 2:**
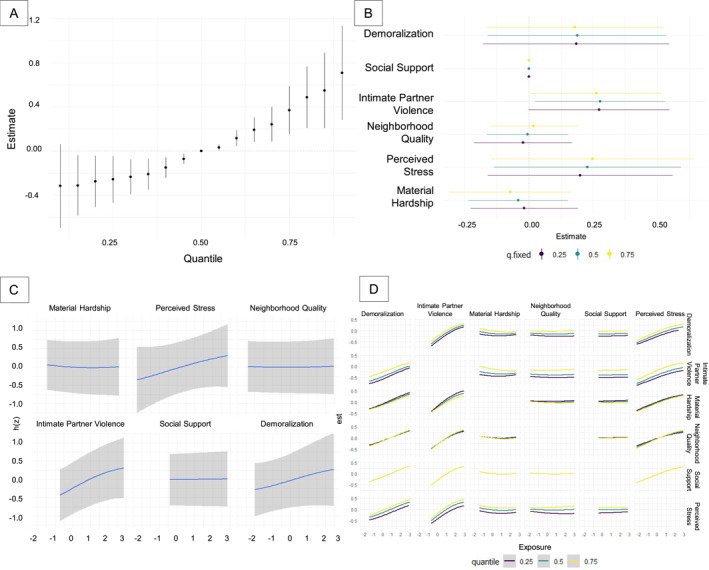
BKMR results of the effect of combined exposure to early‐life familial stressors on the probability of children belonging to the persistently high irritability class. Panel A: overall effects of the stress mixture with 95% CI. The figure shows the estimated change in parent‐reported irritability when stress measures are fixed at particular quantiles from 10th to 90th compared to when all stress measures are fixed at 50th percentile. Panel B: single‐predictor association with irritability. The plot shows the change in irritability with a 95% CI in a single stressor when all other stressors were fixed at either 25th, 50th, or 75th quantile. Panel C: the univariate response functions with 95% confidence bands (shaded areas) for each stressor with all other stressors fixed at the median. Panel D: the response functions for each stressor with one other stressor fixed at either 25th, 50th, or 75th percentile. BKMR, Bayesian kernel machine regression; CI, confidence interval.

BKMR analyses examined associations between the stress mixture and the 2‐class linear irritability trajectory. In the overall stress mixture, more stress associated with higher likelihood of high irritability class membership (CIs in Figure 2) with material hardship, perceived stress, intimate partner violence, and demoralization contributing to the mixture (PIP values in Table 5; Barbieri & Berger, 2004; Nunez et al., 2021). In single variable analyses (when holding effects of other stressors constant), intimate partner violence was positively associated with belonging to the higher irritability class; the shape of the association was linear. In bivariate models, the slopes for each of the stressors were similar at varying levels of other stressors, indicating that the stress variables were additive and did not interact on irritability (Figure 2). The size of the gap between the concentration‐response curves for each stressor indicate additive effects of the stressors on irritability.

**TABLE 5:** Posterior inclusion probabilities (PIP) derived from Bayesian Kernal machine regression models.

Irritability measure included in model	Posterior inclusion probability					
	Material hardship	Perceived stress	Neighborhood quality	Intimate partner violence	Social support	Demoralization
Trajectory	0.578	0.762	0.434	0.977	0.371	0.722
Age 7	0.349	0.81	0.257	1	0.246	0.719
Age 9	0.34	0.534	0.212	0.941	0.231	0.89
Age 11	0.24	0.916	0.199	0.301	0.191	0.501

When examining total irritability scores, the stress mixture associated with greater irritability at all age points. When examining the stressors individually, intimate partner violence was positively and linearly associated with irritability at age 7, whereas perceived stress was positively and linearly associated with irritability at age 11. No stressors independently contributed to age 9 irritability when holding the other stressors constant. Intimate partner violence contributed to the joint mixture effect at ages 7 and 9; perceived stress and demoralization contributed to the joint mixture effect at all timepoints. Bivariate models at all age points confirm that stressor exposures demonstrated additive rather than interactive associations with irritability (Table 5, Supporting Information S1: Figure S5).

Based on their PIP values and consistency of variable selection, perceived stress, intimate partner violence, and demoralization were selected for inclusion in mediation models.

### Causal mediation models

Irritability did not mediate associations between the overall stress mixture or individual stressors and adolescent mental health outcomes (Supporting Information S1: Table S6). Associations of stress and irritability with adolescent outcomes were also not significant (Supporting Information S1: Table S7). Bayesian model comparisons provided converging results.

## DISCUSSION

In a community sample of New York City youth, exposure to intimate partner violence in early childhood predicted higher and more persistent irritability across childhood. Importantly, intimate partner violence is a potentially modifiable target for prevention and marker of risk for children and families in greatest need of intervention. Other stressors—material hardship, perceived stress, and maternal psychological distress—also contributed to irritability persistence, indicating that multiple, co‐occurring stressors shape irritability risk in additive ways and underscoring the importance of preventive screening and early intervention. In contrast to our hypotheses and findings from prior studies (Dougherty et al., 2015; Silver et al., 2024; Sorcher et al., 2022), more persistent childhood irritability did not predict higher anxiety, depression, or externalizing symptoms in adolescence. Our findings thus suggest that there may be important, as yet unknown resilience factors protecting youth from long term effects of early stress exposures.

### Childhood irritability scale

Combined measures of irritability derived from two scales commonly collected in epidemiologic studies indicated the presence of a single general irritability factor at age 7, with increasing dominance of the general factor at ages 9 and 11. Some instrument‐specific variance remained at all time points but was comparatively modest relative to variance accounted for by the general factor. The single latent factor scores demonstrated good test‐retest reliability, moderate stability, and acceptable internal consistency in all 3 waves. This approach allows for studies with extant CBCL and CPRS data to be further leveraged for understanding irritability trajectories.

We observed two classes of irritability development across middle childhood, one smaller class of youth with persistently high irritability and a larger class of youth with persistently low irritability. Prior studies of similarly aged youth with larger samples (*N* > 4000), more ethnoracial diversity, and more waves of data collection have identified four to five latent class solutions representing stable low, stable high, and two to three intermediate classes of irritability that change over time (Bellaert et al., 2025; Riglin et al., 2019; Wiggins et al., 2014; Yu et al., 2022). Possibly, the current smaller and more demographically uniform sample studied over only three‐waves produced fewer classes (Jung & Wickrama, 2008). Further, we observed a higher percentage of youth in the persistently high class (29%) than in prior studies (e.g., 2%–5% Bellaert et al., 2025; Riglin et al., 2019; Wiggins et al., 2014). Notably, only one child demonstrated latent factor z‐scores above 3 standard deviations at age 7 and 11, and no children met this criterion at age 9, suggesting that our findings reflect higher versus lower irritability in our sample rather than clinically elevated irritability. Importantly, although the persistently higher irritability class may not reflect extreme clinical dysregulation, youth with even subclinical elevations in irritability demonstrate considerable functional impairment (Copeland et al., 2015).

### Exposure to intimate partner violence increases risk for elevated irritability trajectory membership

Intimate partner violence was the only stressor that independently associated with irritability class membership. Material hardship, perceived stress, intimate partner violence, and psychological distress all contributed to the joint mixture effect, indicating that when these stressors co‐occur their effects are additive and together increase risk for irritability. Existing biopsychosocial models posit that intimate partner violence may contribute to elevated irritability both biologically, through heightened fear and physiological arousal, and socially, through disruptions to family cohesion or learning maladaptive conflict resolution (Bogat et al., 2023; Márquez et al., 2013; Mead et al., 2010; Tremblay et al., 2018). In animal models, fear induction during the peripubertal period caused aberrant amygdala‐medial orbitofrontal cortex connectivity, which in turn was associated with increased aggression, an animal analogue of irritability (Márquez et al., 2013). Together, these findings support a biological explanation in the biopsychosocial model, suggesting that the neural mechanisms underlying irritability may be preferentially vulnerable to exposure to violence and co‐occurring stressors.

### Exposure to intimate partner violence and maternal perceived stress increase risk for irritability at individual timepoints

Assessing predictors of irritability at individual timepoints further elucidates developmental patterns. In single variable analyses, intimate partner violence was associated with irritability at age 7, and maternal perceived stress was associated with irritability at age 11. In mixtures analyses, intimate partner violence, maternal stress, and psychological distress contributed to irritability at ages 7 and 9, and maternal stress and psychological distress contributed to irritability at age 11. These findings align with the trajectory analyses, suggesting that intimate partner violence exposure in early childhood is compounded by other stressors over time. Consistent with this hypothesis, intimate partner violence is associated with persistent psychological distress among survivors (Chandan et al., 2020; S. J. White et al., 2024). As such, these findings may reflect, in part, the enduring psychological sequelae of intimate partner violence for caregivers, with ongoing maternal stress and distress remaining relevant to children's irritability over time (Chandan et al., 2020; S. J. White et al., 2024). Future longitudinal and mechanistic research should examine associations between stressors on changes in maternal mental health outcomes as well as their bidirectional associations with children's mental health over time.

### Early life stressors did not predict adolescent mental health symptoms

The overall stress mixture and individual stressors were not associated with adolescent mental health symptoms; belonging to the persistently high irritability class did not mediate these associations. Our findings contrast with those of prior studies reporting that psychosocial stress in early life is a predictor of adolescent socioemotional functioning and irritability is a behavioral risk marker for future psychopathology (e.g., Copeland et al., 2014; Silver et al., 2024; Sorcher et al., 2022). Several factors may account for our null findings. Relatively limited variability in irritability and adolescent symptom levels in this community sample may have attenuated associations. Smaller sample size for psychopathology outcomes may have limited power to detect small direct or indirect effects. Future research in larger, epidemiologic samples is needed to better parse these associations.

### Limitations

Our findings should be considered in light of some limitations. First, although our sample is considerably larger than some clinical studies of trauma exposure on trajectories of childhood irritability, our sample size may have been underpowered to detect small effects, particularly in BKMR and mediation analyses. Future studies leveraging large‐scale longitudinal data with repeated measures of irritability will be necessary to disentangle interactive effects between stressors across childhood. Furthermore, our sample is heavily weighted to include people living in disadvantaged contexts, and thus, our findings may not generalize to populations living in advantaged contexts. We note that although we did not have access to a measure of socioeconomic status in this sample, the findings represent a novel contribution to the extant literature as disadvantaged populations have been largely excluded from irritability research. Our epidemiological cohort was recruited through prenatal care clinics, and thus required that mothers be enrolled in prenatal care by the 20^th^ week of pregnancy, potentially biasing our sample. Because our cohort is a community sample that is not weighted for clinical outcomes, our findings are not generalizable to a population of youth with clinically significant mental health concerns. Maternal psychopathology was not assessed and therefore reporter bias could not be directly tested. Youth's baseline psychiatric symptoms were not assessed and therefore we were unable to account for early emerging mental health problems in our models. Moreover, youth self‐reported externalizing symptoms, which may be more accurately assessed by collateral report (Ehrlich et al., 2011; Robinson et al., 2019). Finally, familial stress exposures were self‐reported by mothers during an interview with a research assistant. It is possible that mothers underreported exposure to specific stressors, such as intimate partner violence, due to social desirability or reporter bias.

## CONCLUSIONS

Exposure to familial stressors, and in particular intimate partner violence, contributed to childhood irritability. Our findings elucidate critical time periods and experiences to target when designing prevention and intervention programs for mental health challenges in middle childhood (Romano et al., 2021). When occurring conjointly, material hardship, maternal stress, psychological distress, and intimate partner violence were associated with persistent irritability in children. As such, both intimate partner violence and the accumulation of stress during childhood are important markers for clinical care settings. Future policy priorities should consider the important effects of early life stressors on children and families and how these stressors, including interpersonal violence exposure, contribute to mental and emotional health.

## AUTHOR CONTRIBUTIONS

**Mariah DeSerisy**: Conceptualization; formal analysis; writing—original draft; writing—review and editing. **Huiyu Yang**: Formal analysis; methodology; visualization; writing—original draft. **Jacob W. Cohen**: Conceptualization; methodology; writing—review and editing. **David Pagliaccio**: Conceptualization; methodology; writing—review and editing. **Julie B. Herbstman**: Funding acquisition; project administration; resources. **Bradley S. Peterson**: Funding acquisition; methodology; resources; supervision; writing—review and editing. **Frederica P. Perera**: Funding acquisition; methodology; resources; supervision; writing—review and editing. **Virginia Rauh**: Funding acquisition. **Amy E. Margolis**: Conceptualization; funding acquisition; investigation; methodology; resources; software; supervision; writing—review and editing.

## CONFLICT OF INTEREST STATEMENT

The authors declare no conflicts of interest.

## ETHICAL CONSIDERATIONS

Our study was approved by the Institutional Review Board of Columbia University; initial approval occurred around 11/01/1997 and has been reviewed annually since that date. The most recent annual renewal was approved on 6/24/2026 (Approval Number: Y32M02). Mothers provided informed consent for themselves and their children. Children provided assent beginning at age 7.

## Supporting information

Supporting Information S1

## Data Availability

This study leveraged data from the CCCEH Mothers and Newborns prospective, longitudinal birth cohort. Data is available upon written request.
