# Supplementary material for: Discrete components of stress impact trajectories of childhood irritability but not adolescent mental health outcomes
Source: JCPP Adv. 2026 Aug 9:e70153. Online ahead of print. doi: 10.1002/jcv2.70153 (PMC13454745; doi:10.1002/jcv2.70153)
Supplement: Supplementary file 1 [file JCV2-9999-e70153-s001.docx]

**Discrete Components of Stress Impact Trajectories of Childhood Irritability But Not Adolescent Mental Health Outcomes**

**Supporting Information**

**Appendix S1. Supplementary Methods**

Several published scales were conducted during the standard CCCEH maternal interview. Published scales examine 6 measures of proximal poverty and familial stressors including 8 items examining material hardship, 14 items examining maternal perceived stress, 12 items examining mothers’ experience of intimate partner violence, 8 items examining maternal perceived social support, 32 items examining neighborhood quality, and 27 items examining maternal non-specific psychological distress or demoralization.

The material hardship portion of the interview assessed parent’s level of unmet basic needs in the areas of food, housing, and clothing, e.g., “In the past year has there been a time when you: 1. couldn't afford to buy food?; 2. couldn't afford a place to stay?; 3. couldn't afford gas/electricity?; or 4. couldn't afford clothing?”. Items examining maternal perceived stress were adapted from the Perceived Stress Scale (Cohen et al., 1983) to assess mothers’ current stress level and perceived ability to cope with that stress. Intimate partner violence was examined using items adapted from the short from of the revised Conflict Tactics Scales (Straus et al., 1996), a widely used measure of intimate partner violence in epidemiological cohort studies. This scale measures both physical and psychological violence, including items such as “Caused a serious injury due to a fight you had” or “Yelled and screamed at you.” Perceived social support was measured using items adapted from Cohen’s interpersonal support evaluation list (Cohen et al., 1985, 2000) and examined tangible, emotional, and informational support. Neighborhood quality was examined using items adapted from existing measures of neighborhood quality to assess social order/disorder (0-4 Likert scale; e.g., “Homeless/unemployed people are hanging around”), safety and violence (0-4 Likert scale; e.g., “Letting children go outside during the day/night”), and social cohesion and neighborhood stability (yes/no agreement; e.g., “My neighborhood is a good place to live”). Maternal demoralization was measured using the Psychiatric Epidemiology Research Instrument–Demoralization (PERI-D; Dohrenwend et al., 1980), a 27-item scale measuring eight composite domains of non-specific psychological distress (perceived physical health, sadness, poor self-esteem, dread, anxiety, confused thinking, hopelessness/helplessness, and psychophysiological symptoms).

**Scale Items**

8 items regarding material hardship

1. Think about where you live, the food you eat, and the things you can afford to do and buy. How do you feel about your overall living condition? Would you say?
2. In the last year, has there been a time when you and your family needed food but couldn't afford to buy it?
3. In the last year, has there been a time when you couldn't afford a place to stay, or when you couldn't pay the rent?
4. In the last year, has your gas or electricity been turned off because you couldn't afford to pay the bill?
5. In the last year, have you needed to buy any type of clothing for yourself or your family because you couldn't afford to pay for it?
6. In the last year, has there been a time when you or a member of your family needed medicine or medical care but didn't get the treatment because you couldn't afford it?
7. Do you currently receive Medicaid?
8. Do you currently receive any type of public assistance?

14 items regarding mother’s perceived stress

1. In the last month, how often have you felt that you were unable to control the important things in your life?
2. In the last month, how often have you felt confident about your ability to handle personal problems?
3. In the last month, how often have you felt that things are going your way?
4. In the last month, how often have you felt difficulties were piling up so high that you could not overcome them?
5. You have been upset because of something that happened unexpectedly.
6. You have felt nervous and “stressed”.
7. You have dealt successfully with irritating life hassles.
8. You have felt that you were effectively coping with important changes that were occurring in your life.
9. You have found that you could not cope with all the things you had to do.
10. You have been able to control irritations in your life.
11. You have felt that you were on top of things.
12. You have been angered because of things that happened that were outside of your control.
13. You have found yourself thinking about things that you have to accomplish.
14. You have been able to control the way you spend your time.

12 items regarding experience of intimate partner violence

Many of us have been forced into situations where we have felt scared or uncomfortable. I would like to ask you some questions regarding threats of physical or emotional harm. Since you became pregnant with your child, how often has your partner (Husband, Boyfriend, or any partner you have had since you became pregnant) done any of the following?

1. Threatened to hurt you in any way
2. Caused a serious injury due to a fight you had
3. Used other forms of force during a fight (kicked, pushed, shoved or slapped)
4. Insulted you or embarrassed you in front of others (friends/family/colleagues)
5. Sworn or cursed at you
6. Treated you like an inferior
7. Yelled and screamed at you
8. Monitored and accounted for your whereabouts
9. Been jealous or suspicious of your friends
10. Accused you of having an affair with someone else
11. Interfered in your relationship with other family members
12. Kept you from doing things to help yourself

8 items regarding social support

Now, I would like you to think about people you are close to. Think of the people you live with, your family, and your friends.

1. Do you think there is someone who feels very close to you?
2. Is there someone who you can lean on for support?
3. When you are happy, is there someone you can share it with... Someone who will feel happy simply because you are happy?

I am going to mention several kinds of support most of us need at times. For each, please tell me whether you believe you could get this help if you need it. Do you know

4. Someone that would take you to the doctor, if needed?
5. Someone that would loan you $100 if you needed it?
6. Someone that would help with daily chores if you were sick?
7. Someone that you could talk to about problems in your life?
8. Someone who would watch your children when needed?

32 items about neighborhood quality

The next section contains questions about some troubles you may be experiencing that make your life difficult. We understand that these problems can be very distressing, and are asking these questions to better understand the things in life that make it hard for you to do your job as a mother. I am going to ask you questions about your neighborhood.

Please tell me how often these things happen in your neighborhood

1. Trash is picked up by trash collector
2. Litter or trash stays on the sidewalks or streets
3. Houses and buildings are kept in good repair on the outside
4. Graffiti on buildings and walls
5. Abandoned cars
6. The neighborhood streets are busy with shoppers, mothers with strollers, and children playing
7. Drug dealers make it difficult to feel safe on the street
8. Homeless or unemployed people are hanging around
9. There are vacant or boarded up buildings
10. Gang activity makes it difficult to feel safe
11. Houses and yards not kept up
12. Gunshots and crime make the neighborhood feel unsafe

How worried are you about the following things in your neighborhood?

13. Having property stolen
14. Walking alone during the day
15. Letting children do outside during the day
16. Letting children do outside at the night
17. Being robbed
18. Being murdered

I am going to read several statements about your neighborhood. As I read each one, please answer yes or no.

19. My neighborhood is a good place to live
20. My neighborhood is a good place to raise children
21. The people moving into the neighborhood in the past year or so are good for the neighborhood
22. I would like to move out of this neighborhood
23. When the weather is nice, the people living on my street visit with one another outside
24. The people in my neighborhood visit one another in their homes
25. The people in my neighborhood loan things to one another
26. The people in my neighborhood make sure other’s homes are safe when someone is away
27. On Halloween, most of the children living here go trick-or-treating in my neighborhood
28. People move in and out of my neighborhood a lot
29. There are some children in the neighborhood that I do not want my children to play with
30. Neighbors should mind their own business about other’s children
31. The people moving into the neighborhood lately are bad for the neighborhood
32. Do you ever avoid going outside for safety reasons?

27 items regarding nonspecific maternal distress or demoralization

Now we are going to ask you some questions about your feelings and your state of mind during the past year.

1. During the past year, how often have you felt you were bothered by all different kinds of ailments in different parts of your body…
2. During the past year, how often have you been bothered by feelings of sadness or depression – feeling blue…
3. In general, how satisfied have you been with yourself in the last year...
4. During the past year, how often have you had attacks of sudden fear or panic...
5. During the past year, how often have you felt confident...
6. During the past year, how often have you felt lonely...
7. During the past year, how often have you been bothered by feelings of restlessness
8. During the past year, how often have you felt useless...
9. During the past year, how often have you feared going crazy; losing your mind
10. During the past year, how often have you felt anxious...
11. During the past year, how often have you feared something terrible would happen to you...
12. During the past year, how often have you felt confused and had trouble thinking
13. During the past year, how often have you had trouble concentrating or keeping your mind on what you are doing
14. During the past year, how often have you felt that nothing turns out for you the way you want it to...
15. During the past year, how often have you felt completely hopeless about everything...
16. During the past year, how often have you felt completely helpless....
17. During the past year, how often have you had times when you couldn’t help wondering if anything was worthwhile anymore...
18. During the past year, how often have you been bothered by cold sweats...
19. During the past year, how often have you had trouble with headaches or pains in the head...
20. During the past year, how often has your appetite been poor....
21. In general, if you had to compare yourself with the average woman your age, what grade would you give yourself for the past year....

Are you the kind of person

22. Who feels she has much to be proud of ...
23. Who is the worrying type...
24. Who feels that she is a failure generally, in life...
25. When you have gotten angry in the last year, how often have you felt uncomfortable, like getting headaches, stomach pains, cold sweats and things like that...
26. During the past year, how often have you feared being left all alone or abandoned...
27. During the past year, how often have you been bothered by nervousness, being fidgety or tense.

All stress scales were measured using binomial (Material Hardship) or ordinal response formats (Perceived Stress, Neighborhood Quality, Intimate Partner Violence, Social Support, and Demoralization) and items on some scales (e.g., neighborhood quality, material hardship, social support) included mixed ordered categorical response formats (e.g., both binary and Likert scale items). According to currently accepted recommendations (e.g., Gadermann et al., 2012; Park, 2024), raw Cronbach’s cannot be used for mixed response format scales, which instead require standardized Cronbach’s alpha. This is because raw Cronbach’s alpha is derived from covariances whereas standardized Cronbach’s alpha relies on Pearson correlations between standardized item values. Although feasible for scales with more than 5 Likert scale points (e.g., Demoralization; Rhemtulla et al., 2012), both raw and standardized Cronbach’s alpha underestimate the internal consistency of ordinal scales (Gadermann et al., 2012; Liu et al., 2017). In these cases, currently accepted recommendations support estimating reliability using coefficients derived from polychoric correlations as well as McDonald’s omega (Gadermann et al., 2012; Park, 2024). Specifically, polychoric correlation matrices estimate associations among underlying continuous latent variables and so may be more appropriate than Pearson correlations for ordinal data (Gadermann et al., 2012; Park, 2024). McDonald’s omega, which is based on factor loadings from a latent variable model, estimates the proportion of variance in total scores attributable to the underlying construct rather than measurement error (Gadermann et al., 2012; Park, 2024). However, there is some debate in the field regarding the usefulness of ordinal alpha coefficients (Chalmers, 2018), and so for transparency, we present standardized Cronbach’s alpha, Polychoric Alpha, and McDonald’s Omega in Table S1.

**Childhood Irritability Details for Interpreting Confirmatory Factor Analysis Results**

As described in the main text, Satorra–Bentler scaled chi-square difference test and fit metrics compared the single and two-factor models. Significant chi-square values indicate a significant difference in model fit; fit statistics are then evaluated to determine which model fit the data better (Satorra & Bentler, 2001). In two factor models, psi (Ψ) examines standardized correlations between latent factors, with higher values (e.g., >0.80) suggesting poor factor differentiation (Brown, 2015; Kline, 2023). Fit of bifactor models was evaluated via explained common variance (ECV), Hierarchical Omega, Total Omega, and Percent uncontaminated correlations (PUC) (Reise, 2012; Reise et al., 2013). ECV measures the proportion of shared variance explained by the general factor (i.e., how much variance is explained by the overall latent factor). Hierarchical Omega indicates the amount of reliable variance that is attributable to the general factor (i.e., how much variance in a score reflects a true construct and not measurement error). Total omega reflects the overall reliability attributable to all common factors. Percent uncontaminated correlations (PUC) reflects the proportion of correlations that are not explained by the group factors (i.e., low PUC indicates that subfactors exist, are distinguishable, and are meaningful; Reise, 2012; Reise et al., 2013). According to Finch (2020), to be considered a significantly better model the CFI must improve by more than 0.01, the RMSEA much improve by 0.015 or greater, and the SRMR must improve by 0.03 or greater.

**Analytic Plan**

Mixture modeling approaches have been widely used in the fields of environmental epidemiology to assess unique and shared effects of multiple, often interrelated environmental contaminants on health outcomes (Dominici et al., 2010; Stafoggia et al., 2017). These approaches hold similar promise in the social and biosciences. Many of the same structural factors that confer risk for chemical neurotoxicant exposures, such as marginalization and neighborhood segregation, also confer risk for exposure to social stressors, such as community violence and poverty (Hajat et al., 2015). Furthermore, individual level factors within families, e.g., parental stress, are influenced by structural factors in the built environment, e.g., poverty and pollutant exposures, and contribute to the intergenerational transmission of mental health symptoms via both social (stress process perspective) and biological (genetic, epigenetic, physiological) mechanisms (DeSerisy et al., 2023, 2024; Paananen et al., 2021; Pearlin, 1989). In order to account for these complex interrelationships between predictor variables as well as between the predictor variables and outcomes, robust statistical methods are warranted (Bather et al., 2024; Bobb et al., 2015).

Bayesian kernel machine regression (BKMR) is a robust, non-parametric statistical learning approach that allows for flexible investigation of interrelationships between predictors as well as between predictors and outcomes because it does not require prespecification of interactions between exposure variables (Bobb et al., 2015). This approach provides a distinct advantage over traditional statistical approaches because it can identify high-impact targets for intervention, allowing for the application of strategies that are more likely to produce meaningful benefits both at the individual and public health levels. BKMR has been used successfully by social epidemiologists to examine associations between several individual, social, and structural factors and psychological distress (Bather et al., 2024).

Herein, we use BKMR to test our hypotheses that greater exposure to discrete familial stressors would contribute to higher irritability in children and that exposure to violence would be a particularly salient contributor, over and above other stressors. One BKMR analysis investigated the association of combined exposure to early-life familial stressors on the child’s irritability at each age point. BKMR analysis was performed in R (bkmr; Bobb, 2022). Within the BKMR modeling framework, variable selection is incorporated through posterior inclusion probabilities (PIPs). PIPs provide information about how important a variable is to the model; variables with PIPs close to zero contribute minimally to the model and are interpreted as less important. Credible intervals provide information about the uncertainty, direction, and strength of each variable’s effect; intervals overlapping zero suggest weak or no evidence of an effect.

Each BKMR model provides three outputs. First, BKMR examines the overall mixture effect, in this case the joint effect of the stressors, on the outcome of interest, in this case irritability. Second, the effects of the individual stressors on irritability are examined in single-variable exposure-response functions. Single-variable exposure-response functions summarize the contribution of an individual predictor (i.e., stressor) to irritability score while the remaining predictors are fixed to various quantiles (25th percentile, 50th percentile, and 75th percentile). Single variables whose credible intervals do not cross zero are interpreted as showing evidence of an association with the outcome of interest. Univariate exposure-response curves provide information about the relationship (i.e., linear, parabolic, nonlinear, etc.) between a single exposure and the outcome, with all other exposures fixed to the 50th percentile. Third, bivariate models investigate possible interactions between the stressors by testing the associations between individual stressors and irritability while each of the other stress variables are fixed at various quantiles (in this case, 25th percentile, 50th percentile, and 75th percentile), where larger gaps between the percentile concentration-response (C-R) curves indicate stronger influence of the fixed stressor on irritability.

Of note, BKMR can only be implemented using continuous or binary outcome variables. Thus, in the case that the optimal latent class solution included more than two classes we planned to test the highest irritability class against all the other classes using the BKMR “probit” function (i.e., dummy coded highest irritability class = 1; all other classes = 0).

**Appendix S2. Supplementary Results**

**Details of Participant Demographics**

Correlations between all stress, irritability latent factor, and adolescent symptom scores are shown in Table S2. Table S3 shows longitudinal change is sample size of irritability and adolescent symptom scores. The distribution adolescent symptom scores are shown in Figures S1, respectively.

**Details of Childhood Irritability Scale Development**

Table 3 shows that a two-factor model fit the data slightly better than a single-factor model at all age points (e.g. age 7 CFI .998 vs. 0.996) . However, psi indicated the CBCL and CPRS latent factors were highly correlated at every age (>.85; Table 3), suggesting substantial overlap between the factors. The factors showed acceptable differentiation at age 9 (HTMT = 0.82), borderline differentiation at age 7 (HTMT = 0.85), and poor differentiation at age 11 (HTMT = 0.97). Tables S4-S5 show the bifactor, hierarchical, and two-factor with fixed covariance structure models. At ages 9 and 11, ECV indicated the presence of a strong general factor; ECV was not calculable at age 7 due to instability in the CBCL factor. At ages 9 and 11, hierarchical omega indicated a strong general factor; at age 7, moderate variance was attributable to the general factor. Total omega scores indicate strong reliability of the general factor at every age. At all ages, the PUC was 0.47, representing meaningful variance attributable to underlying subfactors. Identical PUC across ages reflects the identical item structure at every age. The hierarchical model indicated very similar fit to the two-factor structure. When the covariances were fixed (i.e., the factors were forced to be uncorrelated), model fit deteriorated, indicating the CBCL and CPRS factors are measuring related constructs. Correlations between single-factor and global bi-factor scores were r=0.991 at age 9 and r=0.997 at age 11, supporting the notion of a unitary factor solution. Of note, there was variability across goodness of fit indices and HTMT results, with age 9 indices favoring a two-factor model more strongly than a single factor model (i.e., relatively weaker goodness of fit indices when compared to other age points and HTMT demonstrating acceptable differentiation). Despite this, all goodness-of-fit indices fall within commonly accepted ranges (Browne & Cudeck, 1992; Cudeck, 1993; Jöreskog & Sörbom, 1993; Little, 2013; Pavlov et al., 2021). Thus, a single factor solution was selected for use in all analyses.

Internal consistency of single factor irritability composite scores are shown in Table S1. Irritability composite scores were significantly inter-correlated (Figure S3). Across all three time points, single measure ICC was 0.597(95% CI [0.554, 0.639], p < .001) and average-measure ICC was 0. 816 (95% CI [0.788, 0.841], p < .001). The distribution of latent factor scores is shown in Figure S4.

| Table S1. Reliability Statistics for all Measures | | | |
| --- | --- | --- | --- |
| Scale | Pearson’s α (standardized) | Polychoric α^a^ | McDonald’s ω_t_ |
| Early Life Stress Scales | | | |
| Material Hardship^a^ | 0.598 | 0.768 | 0.884 |
| Maternal Demoralization | 0.931 | 0.954 | 0.962 |
| Intimate Partner Violence | 0.923 | 0.965 | 0.975 |
| Social Support^a^ | 0.783 | 0.926 | 0.967 |
| Perceived Stress | 0.784 | 0.808 | 0.869 |
| Neighborhood Quality | 0.873 | 0.933 | 0.951 |
| Adolescent Symptoms | | | |
| Anxiety | 0.875 | 0.935 | 0.944 |
| Depression | 0.844 | 0.901 | 0.926 |
| Externalizing | 0.845 | 0.934 | 0.947 |
| Irritability Scales | | | |
| Age 7 | 0.884 | 0.930 | 0.950 |
| Age 9 | 0.870 | 0.924 | 0.948 |
| Age 11 | 0.874 | 0.927 | 0.943 |
| CPRS only | | | |
| Age 7 | 0.884 | 0.928 | 0.955 |
| Age 9 | 0.857 | 0.917 | 0.943 |
| Age 11 | 0.842 | 0.912 | 0.943 |
| CBCL only | | | |
| Age 7 | 0.638 | 0.775 | 0.781 |
| Age 9 | 0.667 | 0.800 | 0.804 |
| Age 11 | 0.641 | 0.780 | 0.793 |
| Note: ^a^ = tetrachoric correlation matrices were calculated for binary variables | | | |

| Table S2. Correlations between Stress, Irritability Latent Factor, and Adolescent Symptom Scores | | | | | | | | | | | | | |
| --- | --- | --- | --- | --- | --- | --- | --- | --- | --- | --- | --- | --- | --- |
| Variables | 1 | 2 | 3 | 4 | 5 | 6 | 7 | 8 | 9 | 10 | 11 | 12 | 13 |
| 1. Total Mixture Effect | 1*** |  |  |  |  |  |  |  |  |  |  |  |  |
| 2. Material Hardship | 0.278*** | 1*** |  |  |  |  |  |  |  |  |  |  |  |
| 3. Maternal Stress | 0.746*** | 0.341*** | 1*** |  |  |  |  |  |  |  |  |  |  |
| 4. Neighborhood Quality | 0.263*** | 0.146 | 0.245*** | 1*** |  |  |  |  |  |  |  |  |  |
| 5. Intimate Partner Violence | 0.693*** | 0.259*** | 0.18* | 0.173* | 1*** |  |  |  |  |  |  |  |  |
| 6. Maternal Social Support | 0.174* | 0.135 | 0.197** | -0.003 | 0.034 | 1*** |  |  |  |  |  |  |  |
| 7. Maternal Demoralization | 0.71*** | 0.481*** | 0.686*** | 0.212** | 0.232** | 0.223** | 1*** |  |  |  |  |  |  |
| 8. Age 7 Irritability Factor Score | 0.389*** | 0.109 | 0.27*** | 0.085 | 0.301*** | 0.081 | 0.288*** | 1*** |  |  |  |  |  |
| 9. Age 9 Irritability Factor Score | 0.382*** | 0.052 | 0.299*** | 0.052 | 0.19** | 0.114 | 0.291*** | 0.638*** | 1*** |  |  |  |  |
| 10. Age 11 Irritability Factor Score | 0.322*** | 0.088 | 0.26*** | -0.013 | 0.129 | -0.007 | 0.253*** | 0.568*** | 0.651*** | 1*** |  |  |  |
| 11. Adolescent Anxiety Symptoms | -0.018 | -0.086 | 0.022 | -0.122 | -0.062 | -0.231** | -0.021 | 0.045 | 0.084 | 0.052 | 1*** |  |  |
| 12. Adolescent Depression Symptoms | 0.08 | 0.032 | 0.092 | 0 | 0.043 | -0.08 | 0.056 | 0.11 | 0.106 | 0.037 | 0.751*** | 1*** |  |
| 13. Adolescent Externalizing Problems | 0.108 | -0.065 | 0.098 | -0.092 | -0.01 | -0.011 | 0.101 | -0.006 | 0.064 | 0.145 | 0.284** | 0.266** | 1*** |

Note. *=false discovery rate (fdr)-correct p-value<0.05; ** = fdr-corrected p-value <0.01; ***= fdr-corrected p-value <0.001

Table S3. N with available data for each longitudinal irritability and adolescent symptom score.

| Variable | N |
| --- | --- |
| Age 7 Irritability Factor Score | 408 |
| Age 9 Irritability Factor Score | 381 |
| Age 11 Irritability Factor Score | 313 |
| Adolescent Anxiety Symptoms | 203 |
| Adolescent Depression Symptoms | 203 |
| Adolescent Externalizing Problems | 168 |

Table S4. Model fit indices for confirmatory factor analyses

| Model | Χ^2^ | df | CFI | RMSEA | RMSEA CI | SRMR |
| --- | --- | --- | --- | --- | --- | --- |
| Age 7 | | | | | | |
| Single-factor | 62.322 | 35 | 0.996 | 0.044 | 0.025-0.061 | 0.057 |
| Two-factor | 52.824 | 34 | 0.998 | 0.037 | 0.015-0.055 | 0.049 |
| Two-factor fixed covariances | 1962.798 | 35 | 0.745 | 0.368 | 0.354-0.382 | 0.317 |
| Hierarchical | 52.824 | 33 | 0.997 | 0.038 | 0.017-0.057 | 0.049 |
| Bifactor | 26.914 | 25 | 1.000 | 0.014 | 0.000-0.043 | 0.038 |
| Age 9 | | | | | | |
| Single-factor | 82.952 | 35 | 0.992 | 0.060 | 0.043-0.077 | 0.071 |
| Two-factor | 68.703 | 34 | 0.994 | 0.052 | 0.034-0.069 | 0.062 |
| Two-factor fixed covariances | 1603.497 | 35 | 0.731 | 0.343 | 0.329-0.358 | 0.309 |
| Hierarchical | 68.703 | 33 | 0.994 | 0.053 | 0.035-0.071 | 0.062 |
| Bifactor | 44.678 | 25 | 0.997 | 0.046 | 0.023-0.067 | 0.052 |
| Age 11 | | | | | | |
| Single-factor | 56.257 | 35 | 0.996 | 0.044 | 0.021-0.065 | 0.060 |
| Two-factor | 53.819 | 34 | 0.996 | 0.043 | 0.019-0.064 | 0.060 |
| Two-factor fixed covariances | 1910.318 | 35 | 0.621 | 0.414 | 0.399-0.430 | 0.351 |
| Hierarchical | 53.819 | 33 | 0.996 | 0.045 | 0.021-0.066 | 0.060 |
| Bifactor | 26.224 | 25 | 1.000 | 0.013 | 0.000-0.048 | 0.044 |

Note. df=degrees of freedom, CFI=comparative fit index, RMSEA= root mean square error of approximation, CI=confidence interval, SRMR=standardized root mean square residual

| Table S5. Model fit indices for bifactor analyses | | | | | | | | | | |
| --- | --- | --- | --- | --- | --- | --- | --- | --- | --- | --- |
| Age | Χ^2^ | df | CFI | RMSEA | RMSEA CI | SRMR | ECV (General) | ω (Total) | ωH (General) | PUC |
| 7 | 26.914 | 25 | 1.000 | 0.014 | 0.000-0.043 | 0.038 | —* | .91 | .67 | .47 |
| 9 | 44.678 | 25 | 0.997 | 0.046 | 0.023-0.067 | 0.052 | .84 | .90 | .84 | .47 |
| 11 | 26.224 | 25 | 1.000 | 0.013 | 0.000-0.048 | 0.044 | .88 | .90 | .88 | .47 |
| Note. ECV = explained common variance; ω = omega total; ωH = omega hierarchical; PUC = percent uncontaminated correlations. | | | | | | | | | | |

Table S6. Mediation Analyses Irritability Predicting Adolescent Mental Health Symptoms Via Stressors

| Total Mixture Effect | | | | | |
| --- | --- | --- | --- | --- | --- |
| Outcome Variable | Predictor Variable | Mediation Effects | B | 95% Confidence Interval | |
|  |  |  |  | Lower Bound | Upper Bound |
| Adolescent Anxiety Symptoms | High Irritability Class | Direct effect (c′) | -0.153 | -0.572 | 0.274 |
|  |  | Indirect effect (a × b) | 0.151 | -0.025 | 0.329 |
|  |  | Total Effect (c) | -0.002 | -0.404 | 0.398 |
| Adolescent Depression Symptoms | High Irritability Class | Direct effect (c′) | 0.176 | -0.270 | 0.631 |
|  |  | Indirect effect (a × b) | 0.094 | -0.084 | 0.274 |
|  |  | Total Effect (c) | 0.269 | -0.156 | 0.688 |
| Adolescent Externalizing Symptoms | High Irritability Class | Direct effect (c′) | 0.249 | -0.253 | 0.754 |
|  |  | Indirect effect (a × b) | 0.120 | -0.086 | 0.334 |
|  |  | Total Effect (c) | 0.369 | -0.105 | 0.835 |
| Perceived Stress | | | | | |
| Outcome Variable | Predictor Variable | Mediation Effects | B | 95% Confidence Interval | |
|  |  |  |  | Lower Bound | Upper Bound |
| Adolescent Anxiety Symptoms | High Irritability Class | Direct effect (c′) | 0.005 | -0.149 | 0.153 |
|  |  | Indirect effect (a × b) | 0.037 | -0.011 | 0.100 |
|  |  | Total Effect (c) | 0.041 | -0.103 | 0.187 |
| Adolescent Depression Symptoms | High Irritability Class | Direct effect (c′) | 0.084 | -0.070 | 0.236 |
|  |  | Indirect effect (a × b) | 0.027 | -0.022 | 0.087 |
|  |  | Total Effect (c) | 0.111 | -0.041 | 0.256 |
| Adolescent Externalizing Symptoms | High Irritability Class | Direct effect (c′) | 0.094 | -0.074 | 0.263 |
|  |  | Indirect effect (a × b) | 0.034 | -0.018 | 0.103 |
|  |  | Total Effect (c) | 0.128 | -0.036 | 0.296 |
| Intimate Partner Violence | | | | | |
| Outcome Variable | Predictor Variable | Mediation Effects | B | 95% Confidence Interval | |
|  |  |  |  | Lower Bound | Upper Bound |
| Adolescent Anxiety Symptoms | High Irritability Class | Direct effect (c′) | -0.082 | -0.244 | 0.080 |
|  |  | Indirect effect (a × b) | 0.034 | -0.004 | 0.089 |
|  |  | Total Effect (c) | -0.047 | -0.206 | 0.108 |
| Adolescent Depression Symptoms | High Irritability Class | Direct effect (c′) | 0.035 | -0.133 | 0.205 |
|  |  | Indirect effect (a × b) | 0.026 | -0.014 | 0.079 |
|  |  | Total Effect (c) | 0.061 | -0.105 | 0.223 |
| Adolescent Externalizing Symptoms | High Irritability Class | Direct effect (c′) | -0.052 | -0.237 | 0.128 |
|  |  | Indirect effect (a × b) | 0.039 | -0.006 | 0.108 |
|  |  | Total Effect (c) | -0.013 | -0.195 | 0.161 |
| Demoralization | | | | | |
| Outcome Variable | Predictor Variable | Mediation Effects | B | 95% Confidence Interval | |
|  |  |  |  | Lower Bound | Upper Bound |
| Adolescent Anxiety Symptoms | High Irritability Class | Direct effect (c′) | -0.030 | -0.183 | 0.122 |
|  |  | Indirect effect (a × b) | 0.040 | -0.008 | 0.105 |
|  |  | Total Effect (c) | 0.010 | -0.142 | 0.156 |
| Adolescent Depression Symptoms | High Irritability Class | Direct effect (c′) | 0.050 | -0.111 | 0.210 |
|  |  | Indirect effect (a × b) | 0.031 | -0.019 | 0.097 |
|  |  | Total Effect (c) | 0.082 | -0.077 | 0.235 |
| Adolescent Externalizing Symptoms | High Irritability Class | Direct effect (c′) | 0.160 | -0.022 | 0.340 |
|  |  | Indirect effect (a × b) | 0.029 | -0.020 | 0.096 |
|  |  | Total Effect (c) | 0.190 | 0.011 | 0.368 |
| Note: All models included child sex at birth, maternal years of education, maternal age at child birth, and quality of the home environment as covariates. All predictors and covariates were mean-centered and scaled prior to inclusion in analyses. Confidence intervals were generated from simulation distributions derived from 5,000 boot strapped samples. B=standardized coefficients | | | | | |

| Table S7. Direct effects of Stressors or Irritability on Adolescent Mental Health Symptoms | | | | | | |
| --- | --- | --- | --- | --- | --- | --- |
| *Anxiety Symptoms* | | | | | | |
| Predictor | β | Std. Error | Statistic | p-value | FDR-corrected p-value | Bayes Factor |
| Total Mixture Effect | 0.002 | 0.204 | 0.010 | 0.992 | 0.992 | 0.338 |
| Material Hardship | -0.067 | 0.073 | -0.925 | 0.356 | 0.748 | 0.491 |
| Perceived Stress | 0.036 | 0.073 | 0.492 | 0.623 | 0.873 | 0.376 |
| Neighborhood Quality | -0.102 | 0.069 | -1.471 | 0.143 | 0.564 | 0.869 |
| Intimate Partner Violence | -0.047 | 0.078 | -0.601 | 0.548 | 0.873 | 0.396 |
| Social Support | -0.228 | 0.077 | -2.947 | 0.004 | 0.076 | 14.382 |
| Demoralization | 0.007 | 0.076 | 0.091 | 0.928 | 0.992 | 0.339 |
| High Irritability Class | 0.242 | 0.156 | 1.551 | 0.123 | 0.279 | 0.964 |
| Age 7 Irritability | 0.077 | 0.089 | 0.865 | 0.388 | 0.582 | 0.469 |
| Age 9 Irritability | 0.118 | 0.084 | 1.401 | 0.163 | 0.279 | 0.796 |
| Age 11 Irritability | 0.048 | 0.085 | 0.565 | 0.572 | 0.701 | 0.389 |
| *Depression Symptoms* | | | | | | |
| Predictor | β | Std. Error | Statistic | p-value | FDR-corrected p-value | Bayes Factor |
| Total Mixture Effect | 0.274 | 0.214 | 1.283 | 0.201 | 0.603 | 0.722 |
| Material Hardship | 0.041 | 0.076 | 0.543 | 0.588 | 0.873 | 0.403 |
| Perceived Stress | 0.107 | 0.076 | 1.406 | 0.161 | 0.564 | 0.832 |
| Neighborhood Quality | 0.005 | 0.073 | 0.063 | 0.950 | 0.992 | 0.356 |
| Intimate Partner Violence | 0.062 | 0.082 | 0.754 | 0.452 | 0.809 | 0.454 |
| Social Support | -0.079 | 0.083 | -0.946 | 0.345 | 0.748 | 0.522 |
| Demoralization | 0.079 | 0.080 | 0.989 | 0.324 | 0.748 | 0.542 |
| High Irritability Class | 0.235 | 0.165 | 1.429 | 0.155 | 0.279 | 0.856 |
| Age 7 Irritability | 0.162 | 0.094 | 1.733 | 0.085 | 0.279 | 1.294 |
| Age 9 Irritability | 0.144 | 0.089 | 1.624 | 0.106 | 0.279 | 1.104 |
| Age 11 Irritability | 0.040 | 0.089 | 0.442 | 0.659 | 0.719 | 0.386 |
| *Externalizing Symptoms* | | | | | | |
| Predictor | β | Std. Error | Statistic | p-value | FDR-corrected p-value | Bayes Factor |
| Total Mixture Effect | 0.369 | 0.241 | 1.530 | 0.128 | 0.564 | 0.970 |
| Material Hardship | -0.003 | 0.083 | -0.041 | 0.967 | 0.992 | 0.356 |
| Perceived Stress | 0.124 | 0.083 | 1.494 | 0.137 | 0.564 | 0.926 |
| Neighborhood Quality | -0.060 | 0.081 | -0.737 | 0.462 | 0.809 | 0.449 |
| Intimate Partner Violence | -0.012 | 0.089 | -0.139 | 0.890 | 0.992 | 0.358 |
| Social Support | 0.028 | 0.093 | 0.304 | 0.762 | 0.992 | 0.370 |
| Demoralization | 0.189 | 0.089 | 2.134 | 0.034 | 0.361 | 2.491 |
| High Irritability Class | 0.301 | 0.183 | 1.647 | 0.101 | 0.279 | 1.138 |
| Age 7 Irritability | -0.021 | 0.102 | -0.202 | 0.840 | 0.840 | 0.362 |
| Age 9 Irritability | 0.055 | 0.100 | 0.548 | 0.584 | 0.701 | 0.404 |
| Age 11 Irritability | 0.165 | 0.102 | 1.616 | 0.108 | 0.279 | 1.089 |

Note. All models are corrected for child sex, maternal years of education, maternal age at child birth, and HOME total score. Β= estimate; Std: standard; FDR=false discovery rate

Supporting References

Bather, J. R., Robinson, T. J., & Goodman, M. S. (2024). Bayesian kernel machine regression for social epidemiologic research. *Epidemiology (Cambridge, Mass.)*, *35*(6), 735–747.

Bobb, J. F. (2022). *Bayesian Kernel Machine Regression [R package bkmr version 0.2.2]*. https://cran.r-project.org/web/packages/bkmr/index.html

Bobb, J. F., Valeri, L., Claus Henn, B., Christiani, D. C., Wright, R. O., Mazumdar, M., Godleski, J. J., & Coull, B. A. (2015). Bayesian kernel machine regression for estimating the health effects of multi-pollutant mixtures. *Biostatistics (Oxford, England)*, *16*(3), 493–508.

Brown, T. A. (2015). *Confirmatory factor analysis for applied research, second edition* (2nd ed.). Guilford Publications.

Browne, M. W., & Cudeck, R. (1992). Alternative Ways of Assessing Model Fit. *Sociological Methods & Research*, *21*(2), 230–258.

Chalmers, R. P. (2018). On misconceptions and the limited usefulness of ordinal alpha. *Educational and Psychological Measurement*, *78*(6), 1056–1071.

Cohen, S., Kamarck, T., & Mermelstein, R. (1983). A global measure of perceived stress. *Journal of Health and Social Behavior*, *24*(4), 385–396.

Cohen, S., Mermelstein, R., Kamarck, T., & Hoberman, H. M. (1985). Measuring the functional components of social support. In *Social Support: Theory, Research and Applications* (pp. 73–94). Springer Netherlands.

Cohen, S., Underwood, L. G., & Gottlieb, B. H. (2000). *Social support measurement and intervention: A guide for health and social scientists*. *345*. https://doi.org/10.1093/med:psych/9780195126709.001.0001

Cudeck. (1993). of Assessing Model Fit. *Testing Structural Equation Models*. https://books.google.com/books?hl=en&lr=&id=FvIxxeYDLx4C&oi=fnd&pg=PA136&ots=_N_DzWXGAQ&sig=x88l8Kcc8yTFFL_xW6r-HsQZ3KA

DeSerisy, M., Cohen, J. W., Dworkin, J. D., Stingone, J. A., Ramphal, B., Herbstman, J. B., Pagliaccio, D., & Margolis, A. E. (2023). Early life stress, prenatal secondhand smoke exposure, and the development of internalizing symptoms across childhood. *Environmental Health: A Global Access Science Source*, *22*(1), 58.

DeSerisy, M., Salas, L., Akhundova, E., Pena, D., Cohen, J. W., Pagliaccio, D., Herbstman, J., Rauh, V., & Margolis, A. E. (2024). Polycyclic aromatic hydrocarbon exposure effects on trajectories of maternal and adolescent mental health. *Child and Adolescent Psychiatry and Mental Health*, *18*(1), 114.

Dohrenwend, B. P., Shrout, P. E., Egri, G., & Mendelsohn, F. S. (1980). Nonspecific psychological distress and other dimensions of psychopathology. Measures for use in the general population. *Archives of General Psychiatry*, *37*(11), 1229–1236.

Dominici, F., Peng, R. D., Barr, C. D., & Bell, M. L. (2010). Protecting human health from air pollution. *Epidemiology (Cambridge, Mass.)*, *21*(2), 187–194.

Finch, W. H. (2020). Using fit statistic differences to determine the optimal number of factors to retain in an exploratory factor analysis. *Educational and Psychological Measurement*, *80*(2), 217–241.

Gadermann, A. M., Guhn, M., & Zumbo, B. D. (2012). Estimating ordinal reliability for Likert-type and ordinal item response data: A conceptual, empirical, and practical guide. In *Practical Assessment, Research & Evaluation* (Vol. 17, Issue 3). University of Massachusetts Amherst. https://doi.org/10.7275/N560-J767

Hajat, A., Hsia, C., & O’Neill, M. S. (2015). Socioeconomic disparities and air pollution exposure: A global review. *Current Environmental Health Reports*, *2*(4), 440–450.

Jöreskog, K. G., & Sörbom, D. (1993). *LISREL 8: Structural Equation Modeling with the SIMPLIS Command Language*. Scientific Software International.

Kline, R. B. (2023). *Principles and practice of structural equation modeling, fifth edition* (5th ed.). Guilford Press.

Little, T. D. (2013). *Longitudinal Structural Equation Modeling*. Guilford Press.

Liu, Y., Millsap, R. E., West, S. G., Tein, J.-Y., Tanaka, R., & Grimm, K. J. (2017). Testing measurement invariance in longitudinal data with ordered-categorical measures. *Psychological Methods*, *22*(3), 486–506.

Paananen, R., Tuulio-Henriksson, A., Merikukka, M., & Gissler, M. (2021). Intergenerational transmission of psychiatric disorders: the 1987 Finnish Birth Cohort study. *European Child & Adolescent Psychiatry*, *30*(3), 381–389.

Park, C. G. (2024). Implementing alternative estimation methods to evaluate the reliability of Likert-scale instruments. *Women’s Health Nursing (Seoul, Korea)*, *30*(1), 18–25.

Pavlov, G., Maydeu-Olivares, A., & Shi, D. (2021). Using the standardized root mean squared residual (SRMR) to assess exact fit in structural equation models. *Educational and Psychological Measurement*, *81*(1), 110–130.

Pearlin, L. I. (1989). The sociological study of stress. *Journal of Health and Social Behavior*, *30*(3), 241–256.

Reise, S. P. (2012). Invited paper: The rediscovery of bifactor measurement models. *Multivariate Behavioral Research*, *47*(5), 667–696.

Reise, S. P., Bonifay, W. E., & Haviland, M. G. (2013). Scoring and modeling psychological measures in the presence of multidimensionality. *Journal of Personality Assessment*, *95*(2), 129–140.

Rhemtulla, M., Brosseau-Liard, P. É., & Savalei, V. (2012). When can categorical variables be treated as continuous? A comparison of robust continuous and categorical SEM estimation methods under suboptimal conditions. *Psychological Methods*, *17*(3), 354–373.

Satorra, A., & Bentler, P. M. (2001). A scaled difference chi-square test statistic for moment structure analysis. *Psychometrika*, *66*(4), 507–514.

Stafoggia, M., Breitner, S., Hampel, R., & Basagaña, X. (2017). Statistical approaches to address multi-pollutant mixtures and multiple exposures: The state of the science. *Current Environmental Health Reports*, *4*(4), 481–490.

Straus, M. A., Hamby, S. L., BONEY-McCOY, S. U. E., & Sugarman, D. B. (1996). The revised Conflict Tactics Scales (CTS2). *Journal of Family Issues*, *17*(3), 283–316.

Figure Legends

Figure S1. Distribution of adolescent A) anxiety symptoms, B) depression symptoms, and C) externalizing problems scores. Left panel represents z-score distribution. Right Panel shows distribution of raw scores.

Figure S2. Irritability Intra-Item Correlations at A) Age 7 Years, B) Age 9 years, and C) Age 11 Years. Scale color denotes strength of correlations, blue = positive, red = negative. CBCL = Child Behavior Checklist, CPRS = Conners Parent Rating Scale

Figure S3. Correlations between irritability composite scores across age points. Blue indicates positive correlations; darker colors indicate stronger correlations.

Figure S4. Distribution of latent factor Irritability scores

Figure S5. BKMR results of the effect of combined exposure to early-life familial stressors to the child’s irritability at age 1) seven, 2) nine, and 3) eleven. In each, Panel A: overall effects of the stress mixture with 95% CI. The figure shows the estimated change in parent-reported irritability when stress measures are fixed at particular quantiles from 10th to 90th compared to when all stress measures are fixed at 50th percentile. Panel B: single-predictor association with irritability. The plot shows the change in irritability with a 95% CI in a single stressor when all other stressors were fixed at either 25th, 50th, or 75th quantile. Panel C: the univariate response functions with 95% confidence bands (shaded areas) for each stressor with all other stressors fixed at the median. Panel D: the response functions for each stressor with one other stressor fixed at either 25th, 50th, or 75th percentile.
